# Supplementary material for: Exploring Stroke Patients’ Needs after Discharge from Rehabilitation Centres: Meta-Ethnography
Source: Behav Sci (Basel). 2022 Oct 20;12(10):404. doi: 10.3390/bs12100404 (PMC9598696; doi:10.3390/bs12100404)
Supplement: Supplementary file 1 [file behavsci-12-00404-s001.zip › behavsci-1960108-supplementary.pdf]

Supplementary file (A)

Table S1: Example of search carried out in Ovid Medline (1946 to 2021)

	keywords	result
1	Stroke/	107629
2	Cerebrovascular Disease*.mp.	19677
3	Cerebrovascular Disorders/	46868
4	Cerebrovascular accident.mp.	4075
5	brain hemorrhage.mp.	1077
6	Brain Ischemia/	54329
7	CVA.mp.	2530
8	#1 OR #2 OR #3 OR #4 OR #5 OR #6 OR #7	198648
9	limit 8 to (abstracts and english language and humans)	116937
10	Needs Assessment/	31185
11	needs.mp.	343294
12	"Health Services Needs and Demand"/	53907
13	Support.mp.	9569657
14	concern*.mp.	545360
15	demand*.mp.	232715
16	Experience*.mp.	963895
17	wants.mp.	3881
18	Requirement*.mp.	267841
19	Expectation*.mp.	75654
20	#10 OR #11 OR #12 OR #13 OR #14 OR #15 OR #16 OR #17 OR #18 OR #19	10872856
21	limit 20 to (abstracts and english language and humans)	5923259
22	Patient Discharge/	31700
23	Discharge.mp.	173477
24	home.mp.	226767
25	long term.mp.	736793
26	long-term.mp.	736793
27	post-stroke.mp.	8065
28	poststroke.mp.	4775
29	Community.mp.	510621
30	#22 OR #23 OR #24 OR #25 OR #26 OR #27 OR #28 OR #29	1552737
31	limit 30 to (abstracts and english language and humans)	1037599
32	Qualitative Research/	61289
33	Qualitative.mp.	215650
34	Interview/	28779
35	interviews.mp.	186431
36	Focus Groups/	31616
37	focus group.mp.	21348
38	mixed method*.mp.	18138
39	case studies/	2032032
40	#32 OR #33 OR #34 OR #35 OR #36 OR #37 OR #38 OR #39	2407668

41	limit 40 to (abstracts and english language and humans)	1225101
42	#9 AND #21 AND #31 AND #41	1245

Supplementary file (B):

Reasons for rejection:

Studies did not explore needs:

1. Alajbegovic, A.; Djelilovic-Vranic, J.; Alajbegovic, S.; Nakicevic, A.; Todorovic, L.; & Tiric-Campara, M. Post stroke depression. *Medical archives.* **2014,** *68*(1), 47.
2. Beesley, K.; White, J.H.; Alston, M.K.; Sweetapple, A.L.; & Pollack, M. Art after stroke: the qualitative experience of community dwelling stroke survivors in a group art programme. *Disability and rehabilitation*. **2011,** *33*(22-23), 2346-2355.
3. Brookfield, K., ;& Mead, G.; Physical environments and community reintegration post stroke: Qualitative insights from stroke clubs. *Disability & society*. **2016,** *31*(8), 1013-1029.
4. Brunborg, B.; & Ytrehus, S. Sense of well-being 10 years after stroke. *Journal of Clinical Nursing.* **2014,** *23*(7-8), 1055-1063.
5. Buschenfeld, K.; Morris, R.; & Lockwood, S.; The experience of partners of young stroke survivors. *Disability and rehabilitation*. **2009,** *31*(20), 1643-1651.
6. Carlsson, E.; Ehrenberg, A.; & Ehnfors, M. Stroke and eating difficulties: long-term experiences. *Journal of Clinical Nursing.* **2004,** *13*(7), 825-834.
7. Ch'Ng, A. M.; French, D.; & Mclean, N. Coping with the challenges of recovery from stroke: long term perspectives of stroke support group members. *Journal of health psychology*. **2008,** *13*(8), 1136-1146.
8. Chimatiro, G.; and Rhoda, A. "ENVIRONMENTAL BARRIERS TO REINTEGRATION EXPERIENCED BY STROKE CLIENTS POST DISCHARGE FROM A REHABILITATION CENTRE IN MALAWI." *South African Journal of Physiotherapy.* **2014**, 70(1): 18-23.
9. Clarke, P. Towards a greater understanding of the experience of stroke: Integrating quantitative and qualitative methods. *Journal of Aging Studies* **2003,** 17(2): 171-187.
10. Connolly, T. and Mahoney, E. Stroke survivors' experiences transitioning from hospital to home." *Journal of Clinical Nursing* **2018,** 27(21-22): 3979-3987.
11. Croot, E.J.; Ryan,T.W.; Read,J.; Campbell, F.; O'Cathain, A.; & Venables, G. Transient ischaemic attack: a qualitative study of the long term consequences for patients. *BMC family practice.* **2014**, *15*(1), 1-8.
12. Crowe, C.; Coen, R.F.; Kidd, N.; Hevey, D.; Cooney, J.; & Harbison, J. A qualitative study of the experience of psychological distress post-stroke. *Journal of health psychology*. **2016**, *21*(11), 2572-2579.
13. Culler, K.H.; Wang, Y.C.; Byers, K.; & Trierweiler, R. Barriers and facilitators of return to work for individuals with strokes: perspectives of the stroke survivor, vocational specialist, and employer. *Topics in stroke rehabilitation.* **2011,** 18(4), 325-340.

14. da Silva, J.K.; da Silva Carvalho Vila, V. Martins Ribeiro, M. F., & Vandenberghe, L. Survivors' perspective of life after stroke. *Revista Eletrônica de Enfermagem*. **2018,** *18*.
15. Danzl, M.M.; Hunter, E.G.; Campbell, S.; Sylvia, V.; Kuperstein, J.; Maddy, K.; & Harrison, A. "Living with a ball and chain": The experience of stroke for individuals and their caregivers in rural Appalachian Kentucky. *The Journal of Rural Health.* **2013,** *29*(4), 368-382.
16. DiGregorio, T.; and Matthew, J. Interviewing stroke survivors about experiences of their stroke journey." *British Journal of Neuroscience Nursing.* **2020,** 16(Sup2): S16-S17.
17. Donnellan, C.; Martins, A.; Conlon, A.; Coughlan, T.; O'Neill, D.; & Collins, D.R. Mapping patients' experiences after stroke onto a patient-focused intervention framework. *Disability and rehabilitation*. **2013**, *35*(6), 483-491.
18. Doolittle, N.D. *Life after stroke: Survivors' bodily and practical knowledge of coping during recovery*, University of California, San Francisco, **1990.**
19. Dowswell, G.; Dowswell, T.; Lawler, J.; Green, J.; & Young, J. Patients' and caregivers' expectations and experiences of a physiotherapy intervention 1 year following stroke: a qualitative study. *Journal of Evaluation in Clinical Practice.* **2002,** *8*(3), 361-365.
20. Duxbury, S.; DePaul, V.; Alderson, M.; Moreland, J.; & Wilkins, S. Individuals with stroke reporting unmet need for occupational therapy following discharge from hospital. *Occupational Therapy in Health Care.* **2012,** *26*(1), 16-32.
21. Easton, K.L. Post-stroke journey: from agonizing to owning, *Wayne State University* **2001,** 186 p-186 p.
22. Ekstam, L.; Tham, K.; & Borell, L. Couples' approaches to changes in everyday life during the first year after stroke. *Scandinavian journal of occupational therapy*. **2011,** *18*(1), 49-58.
23. Ellis-Hill, C.; Robison, J.; Wiles, R.; McPherson, K.; Hyndman, D.; Ashburn, A.; & On Behalf Of The Stroke Association Rehabilitation Research Centre Team. Going home to get on with life: patients and carers experiences of being discharged from hospital following a stroke. *Disability and rehabilitation.* **2009**, *31*(2), 61-72.
24. Falkenberg, H.K.; Mathisen, T.S.; Ormstad, H.; & Eilertsen, G. "Invisible" visual impairments. A qualitative study of stroke survivorsexperience of vision symptoms, health services and impact of visual impairments. *BMC Health Services Research*. **2020,** *20*(1), 1-12.
25. Falkenberg, H.K.; Mathisen, T.S.; Ormstad, H.; & Eilertsen, G. "Invisible" visual impairments. A qualitative study of stroke survivorsexperience of vision symptoms, health services and impact of visual impairments. *BMC Health Services Research.* **2020**, *20*(1), 1-12.
26. Gilworth, G.; Phil, M., Cert, A.D.; Sansam, K.A.J.; & Kent, R. M. Personal experiences of returning to work following stroke: an exploratory study. *Work*. **2009,** *34*(1), 95-103.
27. Govender, P.; Bricknell, K.; Naidoo, D.; Message, H.; Njoko, S.; & Ayob, Z. 'No one prepared me to go home': Cerebrovascular accident survivors' experiences of community reintegration in a peri-urban context. *African journal of primary health care and family medicine.* **2019,** *11*(1), 1-8.
28. Gustafsson, L.; & Bootle, K. Client and carer experience of transition home from inpatient stroke rehabilitation. *Disability and Rehabilitation.* **2013,** *35*(16), 1380-1386.
29. Hartke, R.J.; Trierweiler, R.; & Bode, R. Critical factors related to return to work after stroke: a qualitative study. *Topics in stroke rehabilitation.* **2011,** *18*(4), 341-351.
30. Hartman-Maeir, A.; Soroker, N.; Ring, H.; Avni, N.; & Katz, N. Activities, participation and satisfaction one-year post stroke. *Disability and rehabilitation.* **2007,** *29*(7), 559-566.

31. Howe, T.J.; Worrall, L.E.; & Hickson, L.M. Interviews with people with aphasia. Environmental factors that influence their community participation. *Aphasiology*. **2008,** *22*(10), 1092-1120.

32. Ing, M.M.; Vento, M. A.; Nakagawa, K.; & Linton, K.F.A qualitative study of transportation challenges among intracerebral hemorrhage survivors and their caregivers. *Hawai'i Journal of Medicine & Public Health.* **2014,** *73*(11), 353.

33. Johnson, M.A. "A qualitative analysis of the lived experiences of African Americans diagnosed with primary stroke. *Dissertation Abstracts International Section A: Humanities and Social Sciences* **2014**, 75(6-A(E)): No Pagination Specified.

34. Leahy, D.M.; Desmond, D.; Coughlan, T.; O'Neill, D.; & Collins, D.R. Stroke in young women: An interpretative phenomenological analysis. *Journal of Health Psychology*. **2016,** *21*(5), 669-678.

35. Lutz, B. J.; Ellen Young, M.; Cox, K. J.; Martz, C.; & Rae Creasy, K. The crisis of stroke: experiences of patients and their family caregivers. *Topics in stroke rehabilitation*. **2011**, *18*(6), 786-797.

36. Mohd Nordin, N.A.; Aziz, N.A.A.; Abdul Aziz, A.F.; Ajit Singh, D.K.; Omar Othman, N.A.; Sulong, S.; & Aljunid, S.M. Exploring views on long term rehabilitation for people with stroke in a developing country: findings from focus group discussions. *BMC health services research.* **2014**,*14*(1), 1-10.

37. O'Connell, B.; Hanna, B.; Penney, W.; Pearce, J.; Owen, M.; & Warelow, P.Recovery after stroke: a qualitative perspective. *Journal of quality in clinical practice.* **2001,** *21*(4), 120-125.

38. Olofsson, A.; Andersson, S.O.; & Carlberg, B. 'If only I manage to get home I'll get better'-Interviews with stroke patients after emergency stay in hospital on their experiences and needs. *Clinical rehabilitation*. **2005,** *19*(4), 433-440.

39. Rhoda, A.; Cunningham, N.; Azaria, S.; & Urimubenshi, G. Provision of inpatient rehabilitation and challenges experienced with participation post discharge: quantitative and qualitative inquiry of African stroke patients. *BMC Health Services Research.* **2015,** *15*(1), 1-9.

40. Röding, J.; Lindström, B.; Malm, J.A.N.; & Öhman, A. Frustrated and invisible--younger stroke patients' experiences of the rehabilitation process. *Disability and rehabilitation.* **2003**, *25*(15), 867-874.

41. Shannon, R.L.; Forster, A.; & Hawkins, R.J. A qualitative exploration of self-reported unmet need one year after stroke. *Disability and rehabilitation*. **2016,** 38(20), 2000-2007.

42. Simeone, S.; Savini, S.; Cohen, M.Z.; Alvaro, R.; & Vellone, E. The experience of stroke survivors three months after being discharged home: A phenomenological investigation. *European Journal of Cardiovascular Nursing*. **2015,** *14*(2), 162-169.

43. Tyagi, S.; Luo, N., Tan, C.S.; Tan, K.B.; Tan, B.Y.; Menon, E.; ... & Koh, G.C.H. Seeking healthcare services post-stroke: a qualitative descriptive study exploring family caregiver and stroke survivor perspectives in an asian setting. *BMC neurology*, **2021.** *21*(1), 1-16.

44. Visvanathan, A.; Mead, G.; Dennis, M.; Whiteley, W.; Doubal, F.; & Lawton, J. Maintaining hope after a disabling stroke: A longitudinal qualitative study of patients' experiences, views, information needs and approaches towards making treatment decisions. *PLoS One*. **2019,** *14*(9), e0222500.

45. White, J. H.; Miller, B.; Magin, P.; Attia, J.; Sturm, J.; & Pollack, M. Access and participation in the community: a prospective qualitative study of driving post-stroke. *Disability and rehabilitation*. **2012,** *34*(10), 831-838.
46. White, J. H.; Gray, K. R.; Magin, P.; Attia, J.; Sturm, J.; Carter, G.; & Pollack, M. Exploring the experience of post-stroke fatigue in community dwelling stroke survivors: a prospective qualitative study. *Disability and rehabilitation.* **2012,** 34(16), 1376-1384.
47. White, J. H.; MacKenzie, L.; Magin, P.; & Pollack, M. R. The occupational experience of stroke survivors in a community setting. *OTJR: Occupation, Participation and Health* **2008**, *28*(4), 160-167.
48. Wiles, R.; Ashburn, A.; Payne, S.; & Murphy, C. Discharge from physiotherapy following stroke: the management of disappointment. *Social science & medicine*. **2004,** *59*(6), 1263-1273.
49. Worrall, L.; Sherratt, S.; Rogers, P.; Howe, T.; Hersh, D.; Ferguson, A.; & Davidson, B. What people with aphasia want: Their goals according to the ICF. *Aphasiology*. **2011,** *25*(3), 309-322.
50. Wottrich, A.W.; Åström, K.; & Löfgren, M. On parallel tracks: newly home from hospital—people with stroke describe their expectations. *Disability and rehabilitation*. **2012,** *34*(14), 1218-1224.

Not relevant stage:

1. Alexandrov, A.W.; Brewer, B. B.; Moore, K.; Grau, C.; Beenstock, D.J.; Cudlip, F.; ... & Alexandrov, A. V. Measurement of patients' perceptions of the quality of acute stroke services: Development and validation of the STROKE perception report. *Journal of Neuroscience Nursing.* **2019,** 51(5), 208-216.
2. Henderson, A.; Milburn, D.; & Everingham, K. Where to from here: patients of a day hospital rehabilitation programme perceived needs following stroke. *Contemporary Nurse*. **1998,** 7(4), 211-216.
3. Ing, M.M.; Linton, K.F.; Vento, M.A.; & Nakagawa, K. Investigation of Stroke Needs (INVISION) Study: stroke awareness and education. *Hawai'i Journal of Medicine & Public Health.* **2015,** 74(4), 141.
4. Nordin, Å.; Sunnerhagen, K.S.; & Axelsson, Å.B. Patients' expectations of coming home with Very Early Supported Discharge and home rehabilitation after stroke-an interview study. *BMC neurology*. **2015,** 15(1), 1-9.
5. Olofsson, A.; Andersson, S.O.; & Carlberg, B. 'If only I manage to get home I'll get better'-Interviews with stroke patients after emergency stay in hospital on their experiences and needs. *Clinical rehabilitation.* **2005,** *19*(4), 433-440.
6. Yeung, S.M.; Wong, F.K.Y.; & Mok, E. Holistic concerns of Chinese stroke survivors during hospitalization and in transition to home. *Journal of advanced nursing.* **2011,** 67(11), 2394-2405.

Not relevant design:

1. Andrew, N.E.; Kilkenny, M.; Naylor, R.; Purvis, T.; Lalor, E., Moloczij, N.; ... & National Stroke Foundation. Understanding long-term unmet needs in A ustralian survivors of stroke. *International Journal of Stroke.* **2014,** 9, 106-112.
2. Boter, H.; Rinkel, G.J.; & De Haan, R.J. Outreach nurse support after stroke: a descriptive study on patients' and carers' needs, and applied nursing interventions. *Clinical Rehabilitation.* **2004,** 18(2), 156-163.
3. Członkowska, A.; Sarzyńska-Długosz, I.; Kwolek, A.; & Krawczyk, M. Evaluation of needs in early post-stroke rehabilitation in Poland. *Neurologia i neurochirurgia polska*. **2006,** 40(6), 471-477.
4. Davoody, N.; Koch, S.; Krakau, I.; & Hägglund, M. Accessing and sharing health information for post-discharge stroke care through a national health information exchange platform-a case study. *BMC medical informatics and decision making.* **2019,** 19(1), 1-16.
5. DePaul, V.G.; Moreland, J.D.; & Dehueck, A.L. Physiotherapy needs assessment of people with stroke following discharge from hospital, stratified by acute functional independence measure score. *Physiotherapy Canada.* **2013,** *65*(3), 204-214.
6. Edwards, D.F.; Hahn, M.G.; Baum, C.M.; Perlmutter, M.S.; Sheedy, C.; & Dromerick, A. W. Screening patients with stroke for rehabilitation needs: validation of the post-stroke rehabilitation guidelines. *Neurorehabilitation and Neural Repair.* **2006,** 20(1), 42-48.
7. Forster, A.; Brown, L.; Smith, J.; House, A.; Knapp, P.; Wright, J. J.; & Young, J. Information provision for stroke patients and their caregivers. *Cochrane database of systematic reviews*. **2012,** (11).
8. Hermans, E.; Anten, H.W.; Diederiks, J.P.; & Philipsen, H. Use of Care by Home-dwelling Stroke Patients during Three Years following Hospital Discharge. *Scandinavian Journal of Caring Sciences*. **1998,** 12(3), 186-190.
9. Hoffmann, T.; & Cochrane, T. What education do stroke patients receive in Australian hospitals?. *Patient education and counselling*. **2009,** *77*(2), 187-191.
10. Hotter, B.; Padberg, I.; Liebenau, A.; Knispel, P.; Heel, S.; Steube, D.; ... & Meisel, A. Identifying unmet needs in long-term stroke care using in-depth assessment and the post-stroke checklist–The Managing Aftercare for Stroke (MAS-I) study. *European Stroke Journal*. **2018,** *3*(3), 237-245.
11. Hung, M.C.; Hsieh, C.L.; Hwang, J.S.; Jeng, J.S.; & Wang, J.D. Estimation of the long-term care needs of stroke patients by integrating functional disability and survival. *PloS one.* **2013,** 8(10), e75605.
12. Ing, M.M.; Linton, K.F.; Vento, M.A.; & Nakagawa, K. Investigation of stroke needs (INVISION) study: Stroke awareness and education. *Hawai'i Journal of Medicine & Public Health.* **2015,** 74(4), 141.
13. Jerome, D.; Dehail, P.; Daviet, J.C.; Lamothe, G.; De Sèze, M.P.; Orgogozo, J.M.; & Mazaux, J.M. Stroke in the under-75S: Expectations, concerns and needs. *Annals of physical and rehabilitation medicine*. **2009,** 52(7-8), 525-537.
14. Johnson, J.; Pearson, V.; & McDivitt, L. Stroke rehabilitation: assessing stroke survivors' long-term learning needs. *Rehabilitation Nursing*. **1997,** 22(5), 243-248.
15. Lincoln, N.B.; Gladman, J.R.F.; Berman, P.; Luther, A.; & Challen, K. Rehabilitation needs of community stroke patients. *Disability and rehabilitation.* **1998,** 20(12), 457-463.

16. Magaard, G.; Wester, P.; Levi, R.; Lindvall, P.; Gustafsson, E., Sedeh, A.N.; ... & Hu, X. Identifying unmet rehabilitation needs in patients after stroke with a Graphic Rehab-CompassTM. *Journal of Stroke and Cerebrovascular Diseases*. **2018,** 27(11), 3224-3235.
17. Martin, B.J.; Yip, B.; Hearty, M.; Marietta, S.; & Hill, R. Outcome, functional recovery and unmet needs following acute stroke. Experience of patient follow up at 6 to 9 months in a newly established stroke service. *Scottish medical journal*. **2002,** 47(6), 136-137.
18. McKevitt, C.; Fudge, N.; Redfern, J.; Sheldenkar, A.; Crichton, S.; Rudd, A.R.; ... & Wolfe, C.D. Self-reported long-term needs after stroke. *Stroke.* **2011,** 42(5), 1398-1403.
19. McKevitt, C.; Fudge, N.; Redfern, J.; Sheldenkar, A.; Crichton, S.; & Wolfe, C. UK stroke survivor needs survey. *London: The Stroke Association*. **2010.**
20. Moreland, J.D.; DePaul, V.G.; DeHueck, A.L.; Pagliuso, S.A.; Yip, D.W.; Pollock, B.J.; & Wilkins, S. Needs assessment of individuals with stroke after discharge from hospital stratified by acute Functional Independence Measure score. *Disability and rehabilitation.* **2009,** 31(26), 2185-2195.
21. O'Halloran, R.; Worrall, L.; & Hickson, L. Stroke patients communicating their healthcare needs in hospital: a study within the ICF framework. *International journal of language & communication disorders.* **2012,** 47(2), 130-143.
22. Op Reimer, W.J.; de Haan, R.S.; Rijnders, P.T.; Limburg, M.; & Van den Bos, G.A. Unmet care demands as perceived by stroke patients: deficits in health care?. *BMJ Quality & Safety*. **1999,** 8(1), 30-35.
23. Padberg, I.; Knispel, P.; Zöllner, S.; Sieveking, M.; Schneider, A.; Steinbrink, J.; ... & Meisel, A. Social work after stroke: identifying demand for support by recording stroke patients' and carers' needs in different phases after stroke. *BMC neurology.* **2016,** *16*(1), 1-8.
24. Patchick, E., et al. "The process and outcomes of six-month reviews in care home settings: are we meeting the needs of stroke survivors in care homes? *Clinical Rehabilitation.* **2018,** 32(10): 1413-1413.
25. Post, M.W.; Visser-Meily, J.M.; & Gispen, L.S. Measuring nursing needs of stroke patients in clinical rehabilitation: a comparison of validity and sensitivity to change between the Northwick Park Dependency Score and the Barthel Index. *Clinical rehabilitation.* **2002,** 16(2), 182-189.
26. Rothwell, K.; Boaden, R.; Bamford, D.; & Tyrrell, P.J. Feasibility of assessing the needs of stroke patients after six months using the GM-SAT. *Clinical Rehabilitation.* **2013,** 27(3), 264-271.
27. Sowtali, S. N., & Harith, S. Educational needs among stroke patients admitted to hospital universiti sains Malaysia: Preliminary findings. *Jurnal Sains Kesihatan Malaysia (Malaysian Journal of Health Sciences)* **2014,** 12(1).
28. Tistad, M.; von Koch, L.; Sjöstrand, C., Tham, K.; & Ytterberg, C. What aspects of rehabilitation provision contribute to self-reported met needs for rehabilitation one year after stroke–amount, place, operator or timing?. *Health Expectations*. **2013,** 16(3), e24-e35.
29. van Veenendaal, H.; Grinspun, D.R.; & Adriaanse, H.P. Educational needs of stroke survivors and their family members, as perceived by themselves and by health professionals. *Patient education and counselling*. **1996,** 28(3), 265-276.

30. Ward, A.; Payne, K.A.; Caro, J.J.; Heuschmann, P.U.; & Kolominsky-Rabas, P.L. Care needs and economic consequences after acute ischemic stroke: the Erlangen Stroke Project. *European journal of neurology.* **2005,** 12(4), 264-267.
31. Yonaty, S.A.; & Kitchie, S. The educational needs of newly diagnosed stroke patients. *Journal of Neuroscience Nursing.* **2012,** 44(5), E1-E9.
32. Ytterberg, C.; Kristensen, H.K.; Tistad, M.; & von Koch, L. Factors related to met needs for rehabilitation 6 years after stroke. *Plos one*. **2020,**15(1), e0227867.

Not relevant population

1. Dalvandi, A.; Ekman, S.L.; Reza Khankeh, H.; Seyed Bagher Maddah, S.; & Heikkilä, K. Rehabilitation experts' experience of community rehabilitation services for stroke survivors in Iran. *Topics in stroke rehabilitation*. **2012,** 19(5), 395-404.
2. Garcia, S.F.; Hahn, E.A.; Magasi, S.; Lai, J.S.; Semik, P.; Hammel, J.; & Heinemann, A.W. Development of self-report measures of social attitudes that act as environmental barriers and facilitators for people with disabilities. *Archives of physical medicine and rehabilitation*. **2015,** 96(4), 596-603.
3. Glickman, L.B. Clients with stroke and non-stroke and their guardians' views on community reintegration status after in-patient rehabilitation. *Malawi Medical Journal.* **2018,** 30(3), 174-179.
4. Hodson, T.; Gustafsson, L.; & Cornwell, P. The lived experience of supporting people with mild stroke. *Scandinavian journal of occupational therapy*. **2020,** 27(3), 184-193.
5. Morris, R. Meeting the psychological needs of community-living stroke patients and carers: a study of third sector provision. *Disability and Rehabilitation.* **2016,** 38(1), 52-61.
6. Visser-Meily, A.; Post, M.; Gorter, J.W.; Berlekom, S.B.V.; Van Den Bos, T.; & Lindeman, E. Rehabilitation of stroke patients needs a family-centred approach. *Disability and rehabilitation.* **2006,** 28(24), 1557-1561.

Studies not in English

1- Kim, N.H.; Kwon, Y.S.; Kim, M.; Lee, K. H.; & Kwak, H.W. A study on the nutritional status, symptoms, and information needs in stroke patients with dysphagia. *Journal of Korean biological nursing science.* **2011,** 13(1), 72-80.

2- Pedreira, L.C.; & Lopes, R.L.M. Vivência do idoso dependente no domicílio: análise compreensiva a partir da historicidade heideggeriana. *Revista Eletrônica de Enfermagem*. **2012,** 14(2), 304-12.

Low quality studies

1. Garrett, D.; & Cowdell, F. Information needs of patients and carers following stroke. *Nursing older people.* **2005,** 17(6).
2. Kamalakannan, S.; Venkata, M.G.; Prost, A.; Natarajan, S.; Pant, H.; Chitalurri, N.; ... & Kuper, H. Rehabilitation needs of stroke survivors after discharge from hospital in India. *Archives of physical medicine and rehabilitation.* **2016,** *97*(9), 1526-1532.
3. McLean, J.; Roper-Hall, A.; Roper-Hall, A.; & Main, A. Service needs of stroke survivors and their informal carers: a pilot study. *Journal of Advanced Nursing.* **1991**, 16(5), 559-564.
4. Vincent, C.; Deaudelin, I.; Robichaud, L.; Rousseau, J.; Viscogliosi, C.; Talbot, L.R.; & Desrosiers, J. Rehabilitation needs for older adults with stroke living at home: perceptions of four populations. *BMC geriatrics*. **2007,** 7(1), 1-17.

Included Studies:

1. Abrahamson, V.; Wilson, P.M. How unmet are unmet needs post-stroke? A policy analysis of the six-month review. *BMC Health Serv. Res.* **2019**, *19*, 480.
2. Chen, L.; Xiao, L.D.; De Bellis, A. First-time stroke survivors and caregivers' perceptions of being engaged in rehabilitation. *J. Adv. Nurs.* **2016**, *72*, 73–84.
3. Dalvandi, A.; Heikkilä, K.; Maddah, S.S.B.; Khankeh, H.R.; Ekman, S.L. Life experiences after stroke among Iranian stroke survivors. *Int. Nurs. Rev.* **2010**, *57*, 247–253.
4. Danzl, M.M.; Harrison, A.; Hunter, E.G.; Kuperstein, J.; Sylvia, V.; Maddy, K.; Campbell, S. "A lot of things passed me by": Rural stroke survivors' and caregivers' experience of receiving education from health care providers. *J. Rural. Health* **2016**, *32*, 13–24.
5. Davoody, N.; Koch, S.; Krakau, I.; Hägglund, M. Post-discharge stroke patients' information needs as input to proposing patient-centred eHealth services. *BMC Med. Inform. Decis. Mak.* **2016**, *16*, 66.
6. Eames, S.; Hoffmann, T.; Worrall, L.; Read, S. Stroke patients' and carers' perception of barriers to accessing stroke information. *Top. Stroke Rehabil.* **2010**, *17*, 69–78.
7. Gard, G.; Pessah-Rasmussen, H.; Brogårdh, C.; Nilsson, Å.; Lindgren, I. Need for structured healthcare organization and support for return to work after stroke in Sweden: Experiences of stroke survivors. *J. Rehabil. Med.* **2019**, *51*, 741–748.
8. Hare, R.; Rogers, H.; Lester, H.; McManus, R.J.; Mant, J. What do stroke patients and their carers want from community services? *Fam. Pract.* **2006**, *23*, 131–136.
9. Harrison, M.; Ryan, T.; Gardiner, C.; Jones, A. Psychological and emotional needs, assessment, and support post-stroke: A multi-perspective qualitative study. *Top. Stroke Rehabil.* **2016**, *24*, 119–125.
10. Hartford, W.; Lear, S.; Nimmon, L. Stroke survivors' experiences of team support along their recovery continuum. *BMC health Serv. Res.* **2019**, *19*, 723.
11. Jones, S.P.; Auton, M.F.; Burton, C.R.; Watkins, C.L. Engaging service users in the development of stroke services: An action research study. *J. Clin. Nurs.* **2008**, *17*, 1270–1279.
12. Lamontagne, M.E.; Richards, C.; Azzaria, L.; Rosa-Goulet, M.; Clément, L.; Pelletier, F. Perspective of patients and caregivers about stroke rehabilitation: The Quebec experience. *Top. Stroke Rehabil.* **2019**, *26*, 39–48.

13. Liddle, J.; Turpin, M.; McKenna, K.; Kubus, T.; Lambley, S.; McCaffrey, K. The Experiences and Needs of People Who Cease Driving After Stroke. *Brain Impair.* **2009**, *10*, 271–281.
14. Lui, M.H.; Mackenzie, A.E. Chinese elderly patients' perceptions of their rehabilitation needs following a stroke. *J. Adv. Nurs.* **1999**, *30*, 391–400.
15. Martinsen, R.; Kirkevold, M.; Sveen, U. Young and Midlife Stroke Survivors' Experiences with the Health Services and Long-Term Follow-Up Needs. *J. Neurosci. Nurs.* **2015**, *47*, 27–35.
16. Mohd Nordin, N.A.; Aziz, N.A.A.; Abdul Aziz, A.F.; Ajit Singh, D.K.; Omar Othman, N.A.; Sulong, S.; Aljunid, S.M. Exploring views on long term rehabilitation for people with stroke in a developing country: Findings from focus group discussions. *BMC Health Serv. Res.* **2014**, *14*, 118.
17. Poulin, V.; Carbonneau, H.; Provencher, V.; Rochette, A.; Giroux, D.; Verreault, C.; Turcotte, S. Participation in leisure activities to maintain cognitive health: Perceived educational needs of older adults with stroke. **2019**, *42*, 4–23.
18. Reed, M.; Harrington, R.; Duggan, Á.; Wood, V.A. Meeting stroke survivors' perceived needs: A qualitative study of a community-based exercise and education scheme. *Clin. Rehabil.* **2010**, *24*, 16–25.
19. Sadler, E.; Daniel, K.; Wolfe, C.D.; McKevitt, C. Navigating stroke care: The experiences of younger stroke survivors. *Disabil. Rehabil.* **2014**, *36*, 1911–1917.
20. Schmitz, M.A.; Finkelstein, M. Perspectives on poststroke sexual issues and rehabilitation needs. *Top. Stroke Rehabil.* **2010**, *17*, 204–213.
21. Shipley, J.; Luker, J.; Thijs, V.; Bernhardt, J. How can stroke care be improved for younger service users? A qualitative study on the unmet needs of younger adults in inpatient and outpatient stroke care in Australia. *Disabil. Rehabil.* **2020**, *42*, 1697–1704.
22. Shook, R.; Stanton, S. Patients' and caregivers' self-perceived stroke education needs in inpatient rehabilitation. *Int. J. Ther. Rehabil.* **2016**, *23*, 278–287.
23. Sumathipala, K.; Radcliffe, E.; Sadler, E.; Wolfe, C.D.; McKevitt, C. Identifying the long-term needs of stroke survivors using the International Classification of Functioning, Disability and Health. *Chronic Illn.* **2012**, *8*, 31–44.
24. Talbot, L.R.; Viscogliosi, C.; Desrosiers, J.; Vincent, C.; Rousseau, J.; Robichaud, L. Identification of rehabilitation needs after a stroke: An exploratory study. *Health Qual. Life Outcomes* **2004**, *2*, 53.
25. White, J.H.; Magin, P.; Pollack, M.R.P. Stroke Patients' Experience with the Australian Health System: A Qualitative Study. *Can. J. Occup. Ther.* **2009**, *76*, 81–89.
26. Wiles, R.; Pain, H.; Buckland, S.; McLellan, L. Providing appropriate information to patients and carers following a stroke. *J. Adv. Nurs.* **1998**, *28*, 794–801.
27. Yeung, E.H.; Szeto, A.; Richardson, D.; Lai, S.H.; Lim, E.; Cameron, J.I. The experiences and needs of C hinese-C anadian stroke survivors and family caregivers as they re-integrate into the community. *Health Soc. Care Community* **2015**, *23*, 523–531.

Supplementary file (C):

Joanna Briggs Institute qualitative critical appraisal checklist

1. Is there congruity between the stated philosophical perspective and the research methodology?
2. Is there congruity between the research methodology and the research question or objectives?
3. Is there congruity between the research methodology and the methods used to collect data?
4. Is there congruity between the research methodology and the representation and analysis of data?
5. Is there congruity between the research methodology and the interpretation of results?
6. Is there a statement locating the researcher culturally or theoretically?
7. Is the influence of the researcher on the research, and vice- versa, addressed?
8. Are participants, and their voices, adequately represented?
9. Is the research ethical according to current criteria or, for recent studies, and is there evidence of ethical approval by an appropriate body?
10. Do the conclusions drawn in the research report flow from the analysis, or interpretation, of the data?

Table S2: Joanna Briggs Institute qualitative critical appraisal checklist

	Abrahamson and Wilson 2019	Chen, Xiao and Bellis 2015	Dalvandi et al. 2010	Danzl et al. 2016	Davoody et al. 2016	Eames et al. 2010	Gard et al. 2019	Garrett, and Cowdell 2005	Hare et al. 2006	Harrison et al. 2016	Hartford, Lear and Nimmon, 2019	Jones et al. 2008	Kamalakannan et al. 2016	Lamontagne et al. 2019	Liddle et al. 2009
1	Y	Y	Y	Y	N	N	N	Y	N	Y	Y	Y	N	Y	Y
2	Y	Y	Y	Y	Y	Y	Y	Y	Y	Y	Y	Y	Y	Y	Y
3	Y	Y	Y	Y	Y	Y	Y	Y	Y	Y	Y	Y	Y	Y	Y
4	Y	Y	Y	Y	Y	Y	Y	Y	Y	Y	Y	Y	Y	Y	Y
5	Y	Y	Y	Y	U	U	U	Y	U	Y	Y	Y	U	Y	Y
6	Y	U	Y	N	N	N	Y	N	Y	Y	Y	Y	N	Y	Y
7	Y	Y	Y	Y	Y	Y	Y	U	Y	Y	Y	Y	N	Y	Y
8	Y	Y	Y	Y	Y	Y	Y	Y	Y	Y	Y	Y	Y	Y	Y
9	Y	Y	Y	U	Y	Y	Y	Y	Y	Y	Y	Y	Y	Y	Y
10	Y	Y	Y	Y	Y	Y	Y	Y	Y	Y	Y	Y	Y	Y	Y
Total	10	9	10	8	7	7	8	8	8	10	10	10	6	10	10

	Lui, and Mackenzie 1999	Martinsen, Kirkevold and Sveen 2015	McLean et al. 1991	Nordin et al. 2014	Poulin et al. 2019	Reed et al. 2010	Sadler et al. 2014	Schmitz and Finkelstein 2010	Shipley et al 2020	Shook and Stanton 2016	Sumathipala et al. 2012	Talbot et al. 2004	Vincent et al. 2007	White, Magin and Pollack 2009	Wiles et al. 1998	Yeung et al. 2015
1	Y	Y	N	N	Y	Y	N	Y	Y	Y	N	Y	Y	Y	Y	N
2	Y	Y	Y	Y	Y	Y	Y	Y	Y	Y	Y	Y	Y	Y	Y	Y
3	Y	Y	Y	Y	Y	Y	Y	Y	Y	Y	Y	Y	Y	Y	Y	Y
4	Y	Y	N	Y	Y	Y	Y	Y	Y	Y	Y	N	Y	Y	Y	Y
5	Y	Y	U	U	Y	Y	U	U	Y	Y	U	Y	Y	Y	Y	U
6	N	N	Y	Y	Y	Y	N	Y	Y	N	N	N	N	Y	Y	N
7	Y	Y	N	Y	Y	Y	Y	Y	Y	Y	Y	Y	Y	Y	U	Y
8	Y	Y	N	Y	Y	Y	Y	Y	Y	Y	Y	N	N	Y	Y	Y
9	Y	Y	N	Y	Y	Y	Y	Y	Y	Y	Y	Y	U	Y	U	Y
10	Y	Y	Y	Y	Y	Y	Y	Y	Y	Y	Y	Y	Y	Y	Y	Y
Total	9	9	4	8	10	10	7	9	10	9	7	7	7	10	8	7

Supplementary file (D):

Table S3

Author	*First order interpretations (what quotes are used to show what participants say)* Second order interpretations (what the authors of articles say).	Codes	Clusters	Themes
Chen et al. 2015	**Readiness to return home**			
	First-time stroke survivors demonstrated low health literacy in stroke and prevention of stoke recurrence. Their needs to learn more about stroke and rehabilitation programmes were usually ignored in busy clinical settings before discharge from the hospital.	The need for information on stroke and recovery programs	Needs for info on stroke	Limited availability and suitability of information
	Participants perceived that staff members were too busy to explain more detailed information to them and inflexible in caregiver training due to time constraint:	Providers time restriction	Factors that influence care experience: accounting for tailoring information need	Care experience
	They never tell me much about the disease. . . .I had very bad experience having a stroke. . .and I said 'Please tell me what I had'. He [the doctor] said, 'Oh I will look into your file'. . .he came back and said, 'Oh you had a LACI'. I said, 'Now what's it short for?', but got no explanation. .	Amount of information given	Adequate information delivery	Limited availability and suitability of information
	.No rehabilitation services information. You got to look up in the phone book and ring them (Adrian, SP8).	The need for rehabilitation programs	Needs for info on rehabilitation program	Limited availability and suitability of information
	I also felt pressured as I was told they had to teach ADL, showering, toileting. . .I had to go in 7 o'clock in the morning. . .I couldn't because I was working then. . .it was very traumatic for me. . . (Eileen, CP6).			
	The caregivers also said that they were not involved in discharge education for stroke survivors with cognitive impairment that required a long-term and considerable effort in rehabilitation and adjustment in their daily activities:	The need for cognitive support	Rehabilitation needs	Adequate care and services

	The only thing I wasn't prepared for was the strategies to manage cognitive changes from the clinical neurologist cos *[because] I had not spoken to her until Bill was walking out of the ward (Jane, CP4).*	Delayed cognitive education	Rehabilitation needs	Adequate care and services
	Moreover, challenges to manage stroke associated cognitive impairment were compounded by other comorbidities that required caregivers to carefully monitor the effect and side effects of treatment:	Need for info on treatment side effects	Needs for info on rehabilitation program	Limited availability and suitability of information
	She [the stroke survivor with cognitive impairment] had no understanding, no insight that she was meant to be on this one [antiplatelet]. . .It certainly has not been explained of medication in the discharge. . .The hospital staff said '. . .we gave you one medication list on discharge' (Jack, CP9).	Needs for info on medication	Needs for info on rehabilitation program	Limited availability and suitability of information
	Participants were not aware of side effects of medications that may cause adverse events and contribute to unexpected hospital readmission:<br><br>*The only thing I thought I was a little bit concerned was that he hasn't had a proper drug review. . .The Baclofen was, probably I wasn't aware that it could've been blocking progress. . . (Rebecca, CP11).*	medication side effects info needs	Needs for info on rehabilitation program	Limited availability and suitability of information
	The participants perceived that knowledge and skills enabling them to maintain activities of daily living, manage ongoing treatments and nursing care for existing chronic conditions were prerequisites for participating in rehabilitation programmes.	ADL training<br>Self-management training	Needs for info on rehabilitation program	Limited availability and suitability of information
	However, the participants were not fully prepared before discharge from hospital:	Discharge education plan	Clear and coordinated plan	Adequate care and services
	...*it was almost like [being] throwing in the deep end. I've never showered anyone in my life before. . .we just sort of muddled through. . .maybe like when you're leaving rehab*	Training of carer	Rehabilitation needs	Adequate care and services

	inpatient, someone probably should go through with the partner of the person about showering, medications. . . (Judy, CP10).			
	These perceptions of not being prepared and engaged to participate in rehabilitation after discharge from hospital clearly reveal the lack of coordinated discharge planning process among the multidisciplinary team that targets first-time stroke survivors and caregivers' educational and training needs. The consequences of maintaining this status quo would be lack of self-care ability and self-efficacy in managing stroke associated chronic conditions, restoring function and preventing complications during rehabilitation. The nurse's involvement as a discharge planner and coordinator for this patient population may be the better intervention to the situation.	The need for clear and coordinated discharge plan<br><br>Consequences of lack of discharge planning<br>Lack of self-efficacy<br>Self-management<br>Restoring function<br>Preventing complication	Clear and coordinated plan<br><br>Clear and coordinated plan	Adequate care and services<br><br>Adequate care and services
	**Coping with care transition**<br>There was a lack of communication and continuity of treatment when the stroke survivors were transferred from one institution to another. Rob expressed his dissatisfaction with unclear care plan when his brother Philip was transferred to a rehabilitation facility. The situation threatened not only the patient's safety but also the outcomes of rehabilitation programme and continuity of care:	The need for effective communication services<br>The needs for continuity of care<br>The need for clear transferred plan	effective communication services<br>rehabilitation needs<br>Clear and coordinated plan	Adequate care and services<br>Adequate care and services<br>Adequate care and services
	. . .at that stage, there was a considerable degree of lack of communication where one didn't know what was happening. . .Philip was first transferred to XX [rehab ward]...so that was the transitional period that was difficult for him. . .(Rob, CP12).	The need for effective communication services	effective communication services	Adequate care and services
	Transition from acute care to rehabilitation is a difficult time for stroke survivors and their family caregivers, who are still			

	grieving the loss of health and independence and trying to adapt to their changed roles in their lives. During the transfer, stroke survivors were at risk of missed care and even a breakdown in the continuum of care caused by inadequate clinical handover, which posed a threat to patient safety and positive rehabilitation outcomes.	Transitional needs<br>Emotional support<br>Adaptational support<br>Missed care<br>Safety<br>Care continuity<br>Rehabilitation outcomes	Discharge experience	Care experience
	Transition from hospital care to home care was a significant step for stroke survivors and caregivers to cope and adjust with daily care activities at home. Stroke survivors and caregivers were facing challenges to adapt to the new role in their life:			Adequate care and services
	I don't have that sounding board. . .it puts an enormous weight of responsibility on me in the fact that I don't have his technical expertise to call upon. . .(Jane,CP4).	Training need for carer	Rehabilitation needs	
	...Sometimes you feel you are left in the limbo...It is just that our bodies both are so tired. . . (Maria, CP3)			Care experience
	...It's unfortunate sometimes I hear Maria, I wish I wasn't here anymore. . . (Henry, SP3)	Patient's Despair	Post discharge experience	Care experiences
	The shortened length of stay in an acute care hospital resulted in most first-time stroke survivors being discharged with ongoing treatment and a level of dependency due to physical and mental impairments. Care transition may be compromised if caregivers were not prepared and supported during the transition:	Shorter hospital care<br>High dependency<br>Compromised transitional needs<br>Unprepared carers	Factors that influence care experience	Care experience
	I was still in the shock of what happened to Sam, back and forth from the hospital...and they said that we had to take the rented house anyway because it was for him to get transitional care. ...The day they sent him home, I was moving to that place [new rented home]. So it was moving and have him in home...he was home only a week and he ended up in XX [residential facility] because I couldn't cope. It was too	DISCHARGE EXPERIENCE<br>Shock<br>Coping issues<br>Cognitive deficits	DISCHARGE EXPERIENCE	

	much...He couldn't understand why he wasn't in his own house (Eileen, CP6).			
	In contrast, provision of education that suits stroke survivors' individual needs, and taking into consideration the home situation and caregivers' emotional endurance, can help smooth the transition from hospital care to home care:<br><br>*I was there [a rehabilitation ward] every day. So I was involved in what he was doing. When we came home, they showed me every- thing. Remember that nurse came out. She told me everything I should be doing. I thought they were very good (Marina, CP8).*	Discharge education/plan:<br>Individualistic<br>Focus on Emotional endurance	Clear and coordinated plan	Adequate care and services
	The variation in the perceptions of care transition is an indicator of a lack of guidelines and a coordinated approach to ensure the continuity of rehabilitation during care transition for all stakeholders involved.	The need for clear and coordinated discharge plan	Clear and coordinated plan	Adequate care and services
	Dealing with fragmented rehabilitation services			
	The participants' perceptions of rehabilitation programmes were stated as fragmented and disorganized. They were compelled to undertake coordination with limited support from health professionals. They perceived these fragmented services as challenging during post-discharge rehabilitation:<br><br>*...No rehabilitation services information. You got to look up in the phone book and ring them...This is how we got onto the XX [rehabilitation centre]...There is no coordination in between...you got to pull them [services] all together . . .so this part coordinates with that. . .I think the basis of good rehab is there now, it just has to be tidied up at the ends (Adrian, SP8).*	REHAB EXPERIENCE<br>Fragmented services<br>Limited professional support<br>Limited information on services	DISCHARGE EXPERIENCE	Care experience

	The participants identified that if they had a person they could talk to regarding services and other concerns it would benefit their post-hospital rehabilitation journey. They suggested that nurses were in an ideal position to coordinate their rehabilitation:	The need for post discharge coordinator	Effective communication services	Adequate care and services
	I just had an idea. A sort of discharge rehab nurse that could ring you perhaps two or three times after you were at home...it would be reassuring even if you didn't have a problem, it would be a link, a transition, intermediate, that would be great (Rebecca, CP11).	Discharge nurse<br><br>Intermediary	Effective communication services	Adequate care and services
	. . .someone to talk to sometimes, I think. Just like a bridge to cross (Michael, SP11).	communicator		
	These participants' suggestion about the nurse's role in post-discharge rehabilitation was based on their experiences in working with nurses in rehabilitation wards in hospitals.			
	The lack of engagement of stroke survivors and caregivers in discussing rehabilitation programmes and in making informed decision of the programmes was evident when the rehabilitative interventions were not delivered consistently to a participant with cognitive impairment that caused unnecessary stress to both the participant and his wife:	Consequences of lack of discharge planning<br>Lack of engagement<br>Stress to carers	Clear and coordinated plan	Adequate care and services
	. . .physiotherapist, occupational therapist and speech therapist. . .they make an appointment. . .but they just come when- ever. . .they overlapped on a couple of occasions. . .in James's case, it got to be structured to a certain degree because he gets confused. . .(Sue, CP2).	Rehab experiences:<br>Uncoordinated professional visits	POST DISCHARGE EXPERIENCE	Care experience
	In contrast, participants also talked about how a motivational interview that health professional used increased their confidence and rehabilitation intensity:	Motivational interviews<br>Improved confidence	Encouragement and motivation	Supportive network

	They asked what my goals were...our driveway at home has got a step down the bottom. I just asked how I should best handle that. . .they talked me through and I tried it at home. . .they've been successful. . .to subconsciously keep you motivated. I look forward to Mondays and Fridays, just to see what's going to happen to me (Andrew, SP10).	Successful Goal-setting	Clear and coordinated plan	Adequate care and services
	When rehabilitation interventions engaged stroke survivors in planning and goal setting, they encouraged stroke survivors to take charge of their rehabilitation, a crucial process to develop their self-care and self-management ability in a long-term recovery.	Engagement in goal setting<br><br>Better self-management	Clear and coordinated plan<br>Rehabilitation needs	Adequate care and services<br>Adequate care and services
	**Uncertainty about ongoing rehabilitation**<br><br>Participants perceived ongoing changing of rehabilitation goals while they experienced different stages of recovery after stroke. However, they had to wait for a period to commence a new rehabilitation programme once they had been discharged from another:	Waiting for rehabilitation	Post discharge experience	Care experience
	Well, each time we were waiting for different packages, it would be about two or three weeks, you know...yeah it affects our life, cos [because] he wasn't getting nothing. We didn't know what is gonna to happen (Eileen, CP6).			
	The lack of continuity of rehabilitation was an indicator of disengaging stroke survivors and caregivers in goal setting and planning rehabilitation programs in a consistent and coordinated way based on stroke survivor's needs in different stages of recovery. In addition, participants were concerned that the interruption of rehabilitation may	Needs for continuity of care<br><br>Rehabilitation delay consequences:<br>jeopardize their recovery	Rehabilitation needs<br><br>Rehabilitation needs	Adequate care and services<br><br>Adequate care and services

	jeopardize their recovery and hinder them from achieving their rehabilitation goals:<br><br>*Philip's primary goal was to get his right arm working as the best he can...so that was a bit of worry early on because we had the two weeks' gap in between being discharged and starting this program and ...it took a week and a half or something before we actually saw the OT [Occupational Therapist] in here other than on the initial session with XX [therapist's name] identifying goals (Rob, CP12).*<br><br>Stroke rehabilitation aims to enable stroke survivors to adapt to their new life with improved physical, emotional and psychological well-being. Stroke survivors' needs change over time. This study revealed the lack of regular assessment and evaluation of stroke survivors' rehabilitation potential, once the planned rehabilitation programme was completed:<br><br>*No automatic follow up on what I am doing. That's the biggest problem. You've got to have some input...There is no follow up unless you do it yourself (Adrian, SP8).*<br><br>Family caregivers expected that ongoing rehabilitation would prevent stroke survivors from physical and cognitive deterioration and stroke associated complications such as falls. Caregivers also believed that engaging in long-term rehabilitation may alleviate caregiver burden. Jack was caring for Mary who had memory loss post stroke. He shared his story about the difficulty he experienced due to a lack of strategies to manage cognitive impairment and rehabilitation for this condition:<br><br>*Mary and I both got iPad. I have asked around the world for Apps on iPad to help her with her memory...so you know,*	hinder them from achieving their rehabilitation goals<br><br>Waiting for rehabilitation<br><br>Lack of follow-up services<br><br>Lack of follow-up services<br><br>NEED FOR ONGOING REHABILITATION<br>Prevent physical deterioration<br>Cognitive decline<br>Reduce caregiver burden<br>Support for cognitively impaired survivors.	Post discharge experience<br><br>Follow up services<br><br>Rehabilitation needs<br><br>Rehabilitation needs	Care experience<br><br>Adequate care and services<br><br>Adequate care and services<br><br>Adequate care support

	cognitive things, I did ask [therapists], I had no information from them (Jack,CP9).<br><br>The participants revealed that inadequate education about how to prevent and manage falls contributed to readmission to hospital for stroke survivors and added stress for caregivers:<br><br>*It [wheelchair] moved with brakes on it and he slipped down there. I didn't even think about the breakage. The next morning I noticed his leg was sticking out to the side like that...he was there [hospital] for three weeks (Eileen, CP6).*<br><br>The above examples indicate that the family caregivers were not well prepared to care for stroke survivors due to lack of ongoing caregiver education and support about management of stroke-related complications.<br>The long-term commitment to caregiver role without adequate caregiver support also resulted in social isolation:<br><br>*I am always here to help him. Assist in the shower, personal care. If I happen to go out, I always set up all his lunch before I go...I am just around most of the time. I don't go out very often (Marina, CP8).*<br><br>The consequences of social isolation without interventions include depressive symptoms, high levels of caregiver burden and low levels of quality of life. Social isolation is also an indicator that the social reintegration based on stroke survivors and caregivers' psychosocial and spiritual needs is largely overlooked in rehabilitation.	Fall prevention info needs<br><br>Training need for carer before discharge	Information needs on stroke<br><br>Rehabilitation needs	Limited availability and suitability of information
